# Perinephric myxoid tumour of fat and IgG4-related disease: when retroperitoneal fat conceals two truths. A case report of a tricky diagnosis

**DOI:** 10.1093/bjrcr/uaaf054

**Published:** 2025-11-18

**Authors:** Maria-Chiara Ambrosetti, Federica Omboni, Francesco Pollastri, Anna Caliò, Giovanni Puppini

**Affiliations:** Radiology Unit, Department of Pathology and Diagnostics, Azienda Ospedaliera Universitaria Integrata of Verona, Verona, 37126, Italy; Radiology Unit, Department of Pathology and Diagnostics, Azienda Ospedaliera Universitaria Integrata of Verona, Verona, 37126, Italy; Rheumatology Unit, Department of Medicine, University of Verona, Verona, 37134, Italy; Section of Pathology, Department of Diagnostic and Public Health, University of Verona, Verona, 37134, Italy; Radiology Unit, Department of Pathology and Diagnostics, Azienda Ospedaliera Universitaria Integrata of Verona, Verona, 37126, Italy

**Keywords:** retroperitoneal fat, IgG4-related disease, perinephric myxoid pseudotumor of fat

## Abstract

The perinephric space is a retroperitoneal compartment that can harbour various types of lesions. Among these, fat-containing lesions represent a specific subset that narrows the differential diagnosis. Perinephric myxoid pseudotumor of fat (PMPFT) is a rare and less-known entity within this category; its imaging features may overlap with other conditions such as well-differentiated liposarcoma and IgG4-related disease. We report the case of a 73-year-old man in whom bilateral perinephric fat-containing lesions were incidentally discovered during an abdominal ultrasound. Further imaging with CT and MRI was performed to better characterise the lesions. Two biopsies were subsequently undertaken; only the second one, performed laparoscopically, yielded diagnostic material, leading to a diagnosis of PMPFT. However, one year later, follow-up CT revealed an increase in size of the left perinephric mass, which also contained more evident soft tissue foci. The patient underwent a third biopsy that demonstrated an IgG4-positive plasma cell infiltrate, introducing a diagnostic dilemma.

## Clinical presentation

**Figure 1.**
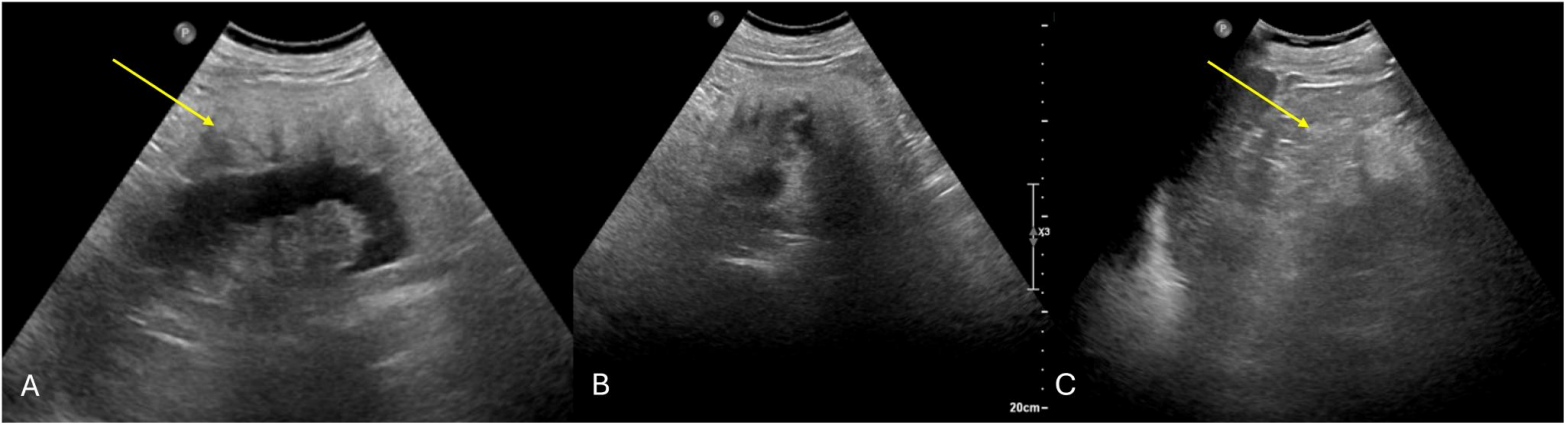
Ultrasound B-mode image. (A) Circumferential hypertrophy and inhomogeneity of the left perirenal adipose tissue (arrow); left kidney maintains normal volume and cortical medullary differentiation. (B, C) The left perirenal adipose tissue hypertrophy extends superiorly (arrow).

A 73-year-old man with no significant comorbidities presented at the urology department of our hospital for a lower urinary tract infection. For this reason, he underwent an abdominal ultrasound (US). At US, kidneys were of regular dimensions and with normal cortico-medullary differentiation. The perirenal adipose tissue of the left kidney was hypertrophic and heterogeneous (Figure 1). For this reason, computed tomography (CT) was suggested.

**Figure 2.**
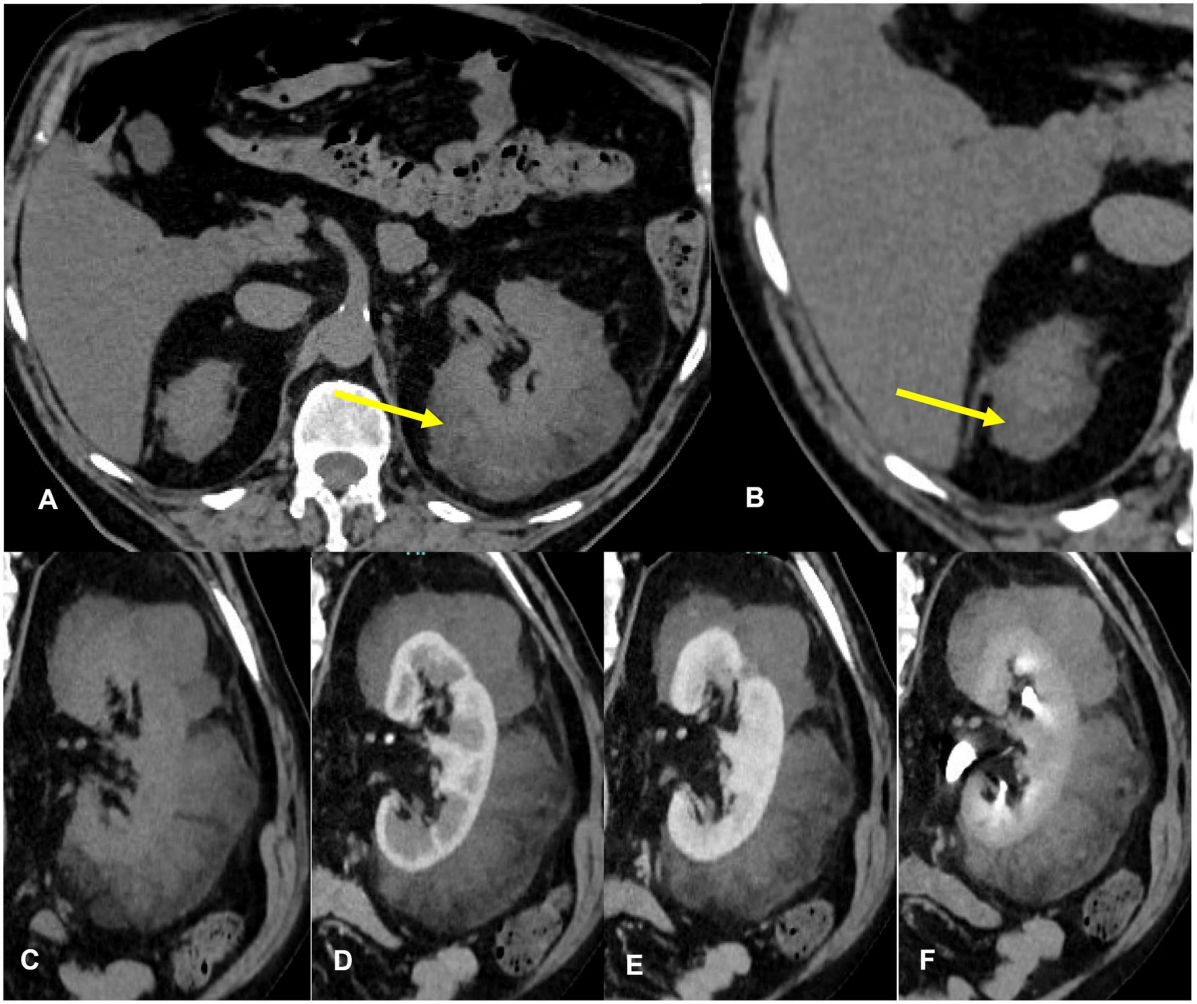
Axial non-contrast CT confirms the presence of a large left perinephric mass, with macroscopic fat foci (-29 HU at ROI measurement, arrow) (A); an analogous smaller lesion can be depicted at the upper pole of right perirenal adipose tissue (detail in B, arrow). Detail of left perinephric lesion that shows definite enhancement (basal value of 16 HU vs delayed phase value of 43 HU): paracoronal CT view of basal (C) and post-contrast arterial phase (D), portal venous phase (E), delayed phase (10 minutes post-contrast injection, F).

At CT a large perinephric mass was depicted, causing anterior dislocation and horizontalisation of the left kidney. The mass contained adipose tissue and large vascularised portions that demonstrated a definite progressive enhancement (>20 HU different from basal to post-contrast delayed phase). An analogous smaller lesion was depicted at the upper pole of the right kidney (Figure 2). No lymphadenomegalies were found in the retroperitoneal space, and there was no free fluid in the abdomen.

**Figure 3.**
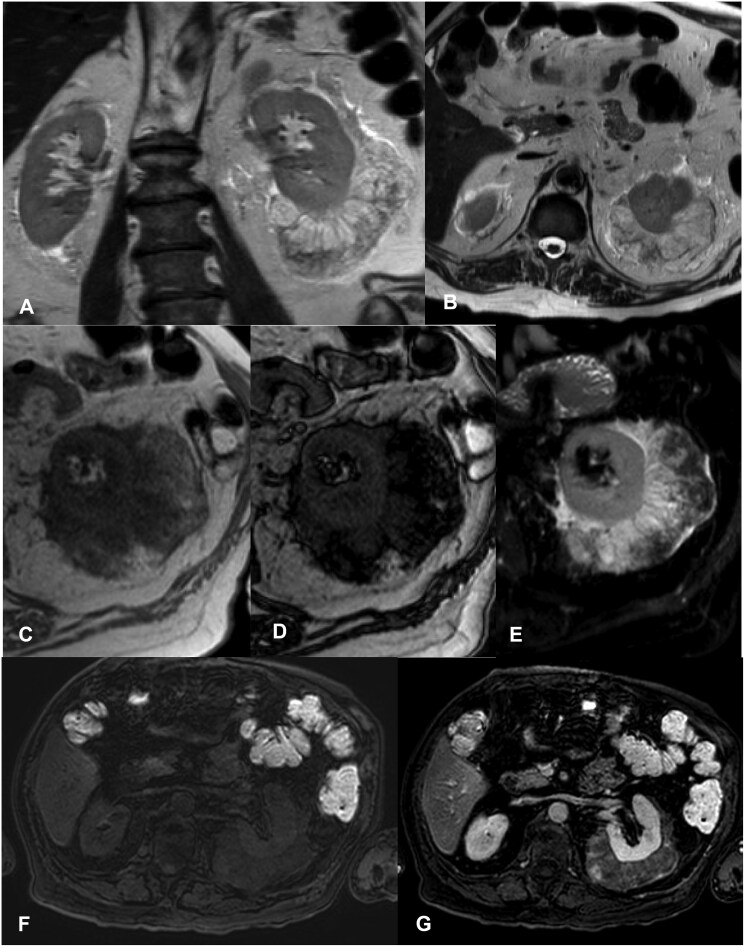
Coronal and axial T2-weighted MR images show the heterogenous hyperintense left perinephric mass (A, B). In- (C) and out-of-phase (D) MR images demonstrate loss of signal of fat foci in opposed phase imaging; comparison with axial SPAIR T2- weighted image at the same level (E). Dixon T1-weight images pre-contrast (F) and post-contrast in the portal venous phase (G) show enhancing parts of the lesion; of note, enhancement is better depicted than in the previous contrast-enhanced CT images.

A Magnetic Resonance (MRI) of the abdomen was done for better characterisation of the perirenal masses. At MRI the perirenal lesion was solid, heterogeneously hyperintense in T1- and T2-weighted images, with large adipose tissue foci depicted with loss of signal in “in and out of phase” sequences (Figure 3). After contrast administration, enhancement of peripheric solid nodules was visible, increasing with time; of note, enhancement was better visible than in the previous CT exam. Smooth hyperintensity on the diffusion sequence was visible.

The lesion localized at the upper lobe of the right kidney had a similar appearance.

At the multidisciplinar board, a biopsy of the mass was indicated to rule out either the benign or malignant nature of the lesion. A CT-guided biopsy was performed, but the material was insufficient for a definitive diagnosis, reporting hypocellular fibromyxoid tissue without necrosis and mitotic activity. Therefore, a video laparoscopic biopsy was performed. At surgery, fibrotic hard perirenal tissue was found, and a fragment of 3 × 2 cm was cut. Histologically, an admixture of mature adipose tissue, myxoid stroma, and bland spindle cells was observed and the final diagnosis of perinephric myxoid pseudotumor of fat (PMPTF) was made.

**Figure 4.**
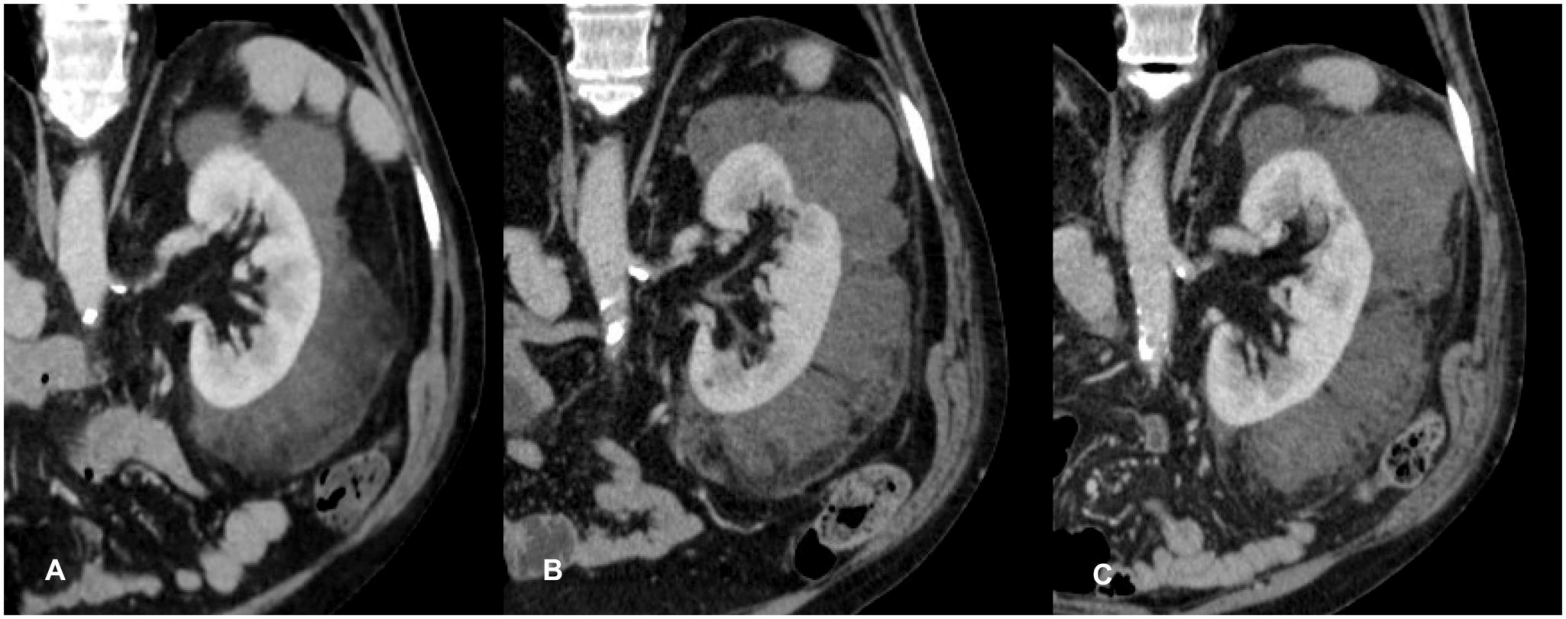
Para-coronal portal venous phase CT, perinephric left lesion at basal (A), 1-year (B) and 2-year time-point (C): lesion demonstrated growth and more heterogeneous features in the follow-up imaging.

After 1 year a CT scan was repeated. In the interval, both the perirenal masses had grown in size and become further heterogeneous with macroscopic foci of fat and solid-enhancing soft tissue components (Figure 4). The 2-year follow-up CT demonstrated the same findings, with further dimensional increase and more evident soft tissue foci (Figure 4).

For this reason, a new biopsy was proposed to investigate the solid thickened foci in the left kidney.

Histology was comparable to the previous specimen, with a mixture of adipose and fibromyxoid tissue, but the presence of IgG4 plasma cells >10x CFI suggested to look for an alternative diagnosis (unfortunately the IgG/IgG4 ratio could not be calculated, even in repeated colorations). Indeed, since the imaging findings were not distinctive of a specific pathology, and suspecting an IgG4 Related Disease (IgG4-RD), additional exams were performed: IgG4 serum concentration was found to be elevated (334 mg/dL, with a normal value under 201 mg/dL), together with total IgG (1560 mg/dL in the first occasion and 1720 mg/dL subsequently, with normal values between 650 and 1600 mg/dL); moreover, total IgE was almost 10 times above the upper normal value, and the CRP was only slightly elevated (7 mg/L). A PET-TC was then performed to exclude other possible sites of disease (and to rule out other systemic diseases), and once excluded, the patient underwent immunosuppressive treatment with a high dose of steroid.

## Discussion

PMPTF is an extremely rare benign fat-containing lesion, arising in the perirenal space. It usually affects male elderly patients (reported mean age of either 59 or 66 years^1^^,^^2^) suffering from chronic kidney disease such as end-stage kidney failure, prior pyelonephritis and various form of nephropathy as diabetic nephropathy which could have a role in the pathogenesis by irritating perinephric fat over time. This entity can also be seen in transplanted kidneys, as previously reported in literature. Of note foci of macroscopic fat are not always visible on imaging exams.^1^

Also, kidney injury is not always present, as in our patient who had no history of kidney disease except for a lower urinary tract infection without clear evidence of pyelonephritis.

PMPTF can present as a solitary or multifocal perinephric mass, sometimes involving the renal sinus, most often containing macroscopic fat that is hyperintense on T2-weighted images, and may show signal dropout on opposed phase imaging. After contrast administration enhancement of the soft tissue components can be depicted and may vary being both homogenous or heterogenous and progressive; enhancement is reported to be better visible at MRI than with CT and in the latter exam lesions can totally lack a visible enhancement.^1^

Furthermore, PMPTF can show slow growth over time,^2^ changing features and becoming even more heterogeneous at imaging follow ups. In the MRI follow up after 1 and 2 years from the diagnosis, both the perirenal lesions of our Patient were significantly grown showing increasing of solid enhancing parts.

All these imaging characteristics overlap with those of other fat-containing lesions, which even occur more frequently, such as extramedullary hematopoiesis, perirenal lipomatosis, fat necrosis, extra-adrenal myelolipoma, perirenal angiomyolipoma or, as a malignant lesion, especially liposarcoma but it can also overlap with non-containing fat lesions such as Erdem Chester disease and IgG4 Related Disease (IgG4-RD)^3^.

In our case the main differential diagnosis was with IgG4 Related Disease (IgG4-RD) with perinephric fat involvement. IgG4-RD is a fibroinflammatory chronic disease that can involve almost every organ in the body. Kidney involvement often presents with solid parenchymal lesions that can either be single or multiple^4^. Among these, pelvic fat and perinephric fat involvement are the rarest manifestation,^5^ with only a few cases reported in case reports and cohort studies of the latter.^6^

According to the 2019 ACR/EULAR classification criteria, IgG4-RD requires both compatible clinical and radiological features and supportive histopathological findings. In our case, the total score was 15, based on serum IgG4 levels upper limit of normal (but less then double), a dense lymphocytic infiltrate and immunostaining showing more than 10 IgG4-positive plasma cells per high-power field (HPF). The diagnostic threshold according to the classification criteria is 20 points,^7^ however, perinephric fat involvement is not currently included in the imaging criteria for IgG4-RD. If it had been considered, both in terms of renal involvement and retroperitoneal involvement the diagnostic threshold might have been reached. However, it is important to note that the ACR/EULAR classification system does not show diagnostic criteria, but classification criteria and thus has high specificity but somewhat lower sensitivity (approximately 82%), meaning that certain true cases may go unclassified.

In addition to these features, the patient exhibited slow progression of the mass, characteristic of IgG4-RD too,^7^ and did not display other signs suggestive of hematologic malignancy or other more aggressive solid tumours.

In our clinical scenario, since the radiological characteristics were not specific and compatible with the diagnosis of both PMTPF and IgG4-RD, the second histology was crucial for the diagnosis. It showed a lymphoplasmacytic infiltrate and positive IgG4 staining, and it ruled out another possible differential diagnosis, well-differentiated liposarcoma, based on both the histological pattern and the absence of MDM2 amplification. From that point on, considering the possibility of IgG4-RD, other compatible laboratory findings were looked for, such as inflammatory markers, elevated levels of IgG, IgG4, and IgE and complement consumption.^8^^,^^9^ Although we did not find certain suggestive features, such as complement consumption or storiform fibrosis on histological examination, we did observe typical findings such as elevated IgG, IgG4, and IgE levels, along with only a mild increase in CRP. These findings led us to hypothesize an early form or a myxoid variant of IgG4-RD.^10^

Correct diagnosis of a perinephric mass can be challenging, and clinical, radiological and anatomopathological information should be evaluated to make a correct final diagnosis. All the information, if alone, is not enough to make a diagnosis, but the combination of them gives the clue. At histology, the presence of lymphoplasmacitic infiltrate and IgG4 at the immunostaining and MDM2 mutation are important considerations in the differential diagnosis. Of note, even if IgG4-related disease guideline diagnosis can be made even without histology, biopsy is often required to make a final diagnosis for these entities.

On the other hand, radiologists evaluating perirenal fat-containing lesions should consider PMPTF as a potential differential diagnosis to provide appropriate clinical management, mostly in the clinical setting of a patient with chronic kidney disease and circumferential perinephric disease with both lipomatous and soft tissue contents as proposed by Broski et al.^1^

Few cases are reported in the literature of both these pathologies, but never reporting multimodality imaging during follow-up of 2 years.

## Learning points

- The perirenal space, as a part of the retroperitoneum, can harbour a wide range of lesions, malignant and benign.
- Perinephric myxoid pseudotumor of fat is one of the benign fat-containing lesions that can arise in the perirenal space, probably the rarest, and at imaging can resemble both other benign fat-containing lesions and malignant entities such as liposarcoma
- IgG4-RD is a fibroinflammatory chronic disease that can involve almost every organ in the body. Kidney involvement often presents with solid parenchymal lesions that can either be single or multiple. Among these, pelvic fat and perinephric fat involvement are the rarest manifestation
- Differential diagnosis between perinephric masses, also given their rarity, can be challenging, and both clinical, radiological and anatomopathological information should be evaluated to make a correct final diagnosis
